# The Role of Silicone Oil in the Surgical Management of Endophthalmitis: A Systematic Review

**DOI:** 10.3390/jcm11185445

**Published:** 2022-09-16

**Authors:** Fabrizio Sinisi, Marco Della Santina, Pasquale Loiudice, Michele Figus, Giamberto Casini

**Affiliations:** 1 Department of Surgical, Medical and Molecular Pathology and Critical Care Medicine, University of Pisa, 56124 Pisa, Italy; 2 Complex Operative Ophthalmology Unit, “F. Lotti” Hospital, 56025 Pontedera, Italy

**Keywords:** antimicrobic activity, endophthalmitis, pars plana vitrectomy, silicone oil, toxicity

## Abstract

(1) Background: We aimed to systematically review the current literature to evaluate if in patients with postoperative endophthalmitis treated with pars plana vitrectomy, silicone oil tamponade could provide a useful contribution to the control and eradication of infection and if it could influence anatomical recovery and functional outcome. (2) Randomized controlled trials, cross-sectional studies, case series, and case reports published in the English language in peer-reviewed journals were included. No restriction was placed based on the study location. We used medical subject headings (MeSH) and text words. We searched MEDLINE (OVID and PubMed), Google Scholar, ISI Web of Science (Thom-on-Reuters), and the Cochrane Library (Wiley) from January 1995 to the present. To ensure literature saturation, we scanned the reference lists of included studies or relevant reviews identified through the search. Risk of Bias was assessed using the Newcastle-Ottawa scale for longitudinal studies and Cochrane risk-of-bias tool for randomized trials. (3) Results: abstracts of 75 articles were selected for full-text reading; after full-text reading, 44 articles were taken into consideration in the systematic review. 5 out of 7 in vitro experimental studies demonstrated antimicrobial activity against different species of bacteria and fungi. The use of SO as endotamponade associated with PPV led to better visual acuity and a lower rate of retinal detachment and the need for additional surgery. (4) Conclusions: Silicone oil reduces the risk of postoperative retinal detachment, especially in case of undetected retinal breaks, produces compartmentalization of the eye, may lead to early visual recovery, allows laser photocoagulation, prevents severe postoperative hypotony and has antimicrobic activity due to an inhibitory effect for several species of pathogens. Concerns regarding possible toxic effects on the retina and optic disc, compartmentalization and impaired washout of pathogen toxins have been reported. It may also influence intravitreal antibiotic distribution and clearance.

## 1. Introduction

Endophthalmitis represents one of the biggest diagnostic and therapeutic challenges in modern ophthalmology. It threatens all forms of intraocular surgery from intravitreal injections to corneal transplants and vitrectomies and it is discussed on all surgical consent forms.

The Endophthalmitis Vitrectomy Study (EVS) was a randomized clinical trial conducted in the United States between 1990 and 1995 [1]. The purpose of this landmark study was to investigate the role of initial pars plana vitrectomy (PPV) and the benefit of intravenous antibiotics for the treatment of postoperative bacterial endophthalmitis.

The EVS concluded that early vitrectomy should be recommended only for cases with visual acuity of light perception or lower on presentation; however, with the technical advances in vitrectomy, in the following years, many authors reported better results using such a technique in patients with visual acuity higher than light perception [2].

The 2013 European society of cataract and refractive surgeons’ guidelines for the prevention and treatment of endophthalmitis after cataract surgery considered a complete vitrectomy and subsequent injection of intravitreal antibiotics as the “gold standard” treatment in case of acute postoperative endophthalmitis [3].

Another debated topic is whether a complete PPV should be followed with a silicone oil (SO) injection given the higher risk of development of retinal breaks and consecutive retinal detachment after vitreous removal.

Interestingly, according to some authors, SO could exert a bacteriostatic activity helping therefore in infection control and preventing the development of reinfection.

The authors aimed to review the current literature concerning the role of SO injection after PPV for the management of postoperative endophthalmitis.

## 2. Materials and Methods

In this work, we adhered to the Preferred Items for Systematic Reviews and Meta-Analyses (PRISMA) guidelines [4]. The protocol was registered on OSF Registries (Registration DOI: 10.17605/OSF.IO/8ZTQJ).

### 2.1. Eligibility Criteria: Participants, Interventions, Comparators, and Outcomes (PICO)

Randomized controlled trials, cross-sectional studies, case series, in vitro experimental studies and case reports published in the English language in peer-reviewed journals, discussing the treatment of endophthalmitis with PPV and SO injection was included. Articles published before 1995 were excluded. No restriction was placed based on the study location.

The objectives of our study were to systematically review the current literature to evaluate the role of silicone oil in patients with postoperative endophthalmitis treated with pars plana vitrectomy and silicone oil tamponade. The proposed review answered the following questions: does silicon oil provide a useful contribution to the control and eradication of infection? Does the use of silicone oil influence the visual recovery and the final visual outcome?

**Figure 1:**
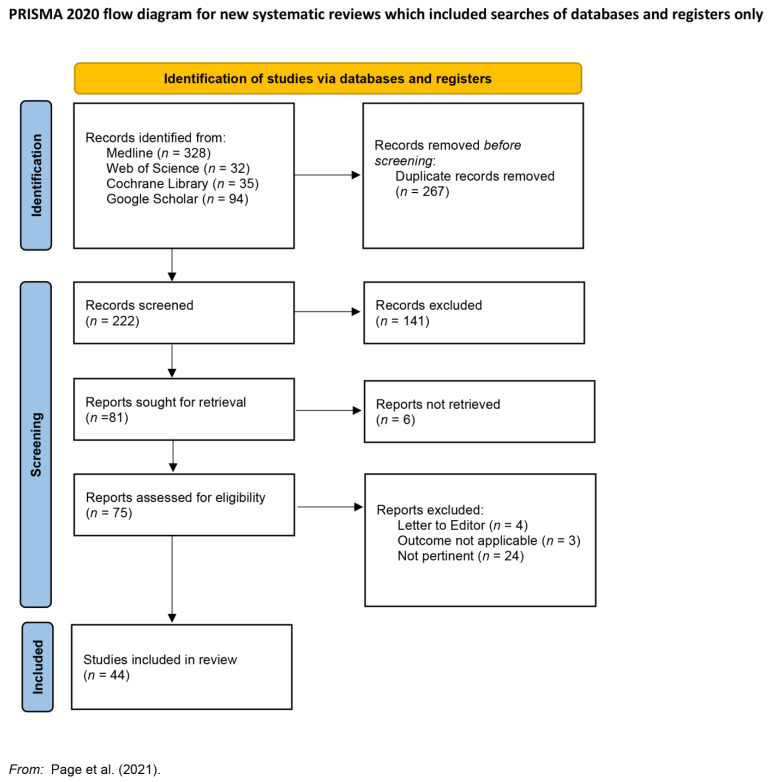
Preferred Items for Systematic Reviews and Meta-Analyses (PRISMA) flow diagram; adapted from Reference [4].

Figure 1 summarizes the PRISMA flow diagram (Figure 1).

### 2.2. Search Strategy

Literature search strategies were developed using medical subject headings (MeSH) and text words. We searched MEDLINE (OVID and PubMed), Google Scholar, ISI Web of Science (Thom-on-Reuters), and the Cochrane Library (Wiley) from January 1995 to March 2022. We used various combinations of key terms related to “endophthalmitis”, “silicone oil” and “pars plana vitrectomy.” Two of the authors (F.S. and M.D.S.) independently performed the research, screening for eligibility. They independently extracted the data using pre-determined forms. Research records were compared to eliminate duplicates. Discrepancies were resolved by agreement between the reviewers or with a third, experienced reviewer (M.F.). Data extracted from every study will include the last name of the first author, year of publication, study design, sample size, purpose, anatomical outcome, visual outcome, and pathogens studied. Outcomes will be the following: antimicrobial activity of silicone oil, anatomical outcome, and visual outcome.

### 2.3. Risk of Bias and Quality of Evidence Assessment

The risk of bias was assessed using the Newcastle-Ottawa scale for longitudinal studies, the Cochrane risk-of-bias tool for randomized trials and the Joanna Briggs Institute (JBI) Critical Appraisal Checklist for Case Series/case reports [5,6]. The Quality of Evidence was assessed using the Grading of Recommendations Assessment, Development and Evaluation (GRADE) system [7]. Thresholds for converting the Newcastle-Ottawa scales are as follows: good quality = above 6 stars; fair quality = 4 to 6 stars; poor quality = 0 to 3 stars [8].

## 3. Results of Searches

In total, 489 records were retrieved from multiple databases including MEDLINE (328), ISI Web of Science (32) Cochrane Library (35), and Google Scholar (94). After removing duplicates (267 records), 222 records were included in the screening process.

Abstracts of 75 articles published in the English language in peer-reviewed journals from January 1995 (conclusion of the Endophthalmitis vitrectomy study—EVS) to March 2022 whose topic was inherent to the surgical treatment of Endophthalmitis with vitrectomy and silicone oil injection were selected for full-text reading. Finally, after full-text reading, 44 articles were taken into consideration in the systematic review.

Of these, 7 were in vitro experimental studies [9,10,11,12,13,14,15], 10 were case reports [16,17,18,19,20,21,22,23,24,25], 17 were case series [26,27,28,29,30,31,32,33,34,35,36,37,38,39,40,41,42], 4 were randomized trials [43,44,45,46] and 6 were retrospective interventional studies [47,48,49,50,51,52].

In in vitro experimental studies, bacterial growth was considered in terms of Colony-forming unit (CFU) or with the onset of inhibition halo. 5 out of 7 studies demonstrated antimicrobial activity against different species of bacteria and fungi.

The use of SO as endotamponade associated with PPV led to better visual outcomes in 3 of the 4 randomized clinical trials. These functional improvements appeared more significant in traumatic subgroups [43]. In all the randomized studies, post-operative retinal detachment rate was chosen as an anatomical outcome. In patients who received SOI, the incidence of retinal detachment ranged between 0% and 6.2% and was significantly lower than in control groups (25.5–76.6%).

In retrospective studies, case series and case reports, functional outcomes were homogenously defined as best-corrected visual acuity improvements whereas anatomical success was heterogeneously identified by different Authors as postoperative retinal detachment rate, post-operative hypotony, the appearance of retinal atrophy, intravitreal injection rate, inflammation control and evisceration rate.

Given the low incidence of the disease, most of the evidence was derived from case reports and case series. The Authors chose to include these types of studies and evaluated them according to the JBI critical evaluation tool (Tables S5 and S6). All case series did not report the demographic information relative to the centres where the research was conducted; this parameter was not considered relevant to the quality of evidence considering that this review mainly focused on treatment strategies of endophthalmitis independently from the demographic area where the pathology occurs.

Two out of 4 RCTs had a low risk of bias [43,45]; higher scores of quality of evidence (GRADE system) were observed in prospective randomized trials [43,44,45,53], and retrospective studies with clearly defined control groups [49,50,54].

A synthesis of the results was displayed in Table 1 and Table 2.

Considering the type and quality of evidence, the Authors chose to report a qualitative analysis, detailed issue-by-issue in a narrative fashion for the heterogeneity of available data and the design of the studies.

## 4. Definition

Endophthalmitis is an inflammatory process involving the internal structures of the eye mainly caused by exogenous agents such as bacteria, mycetes and occasionally parasites which may penetrate the eye in the intraoperative or postoperative phase, after eye injuries or may spread from ocular surface infections.

Postoperative endophthalmitis can be classified as acute, whether the infection is developed within 6 weeks from the intraocular procedure, or chronic.

Rarely, in endogenous endophthalmitis, the infectious agent reaches the eye through the bloodstream; this usually occurs in patients with risk factors such as immunosuppression or intravenous drug abuse.

### Clinical Features

Endophthalmitis is usually defined by severe inflammation of the ocular tissues and fluids characterized clinically by a combination of signs and symptoms including ocular pain, decreased vision, eyelid oedema, conjunctival congestion, chemosis, anterior segment inflammation, hypopyon, vitritis, and decreased red reflex.

## 5. Epidemiology and Causative Agents

The incidence of postoperative endophthalmitis has sensibly decreased over the years. Most cases of endophthalmitis are exogenous, and organisms are introduced into the eye via trauma, surgery, or ocular surface infections. Endogenous endophthalmitis occurs when the eye is seeded via the bloodstream [57].

The rate of occurrence ranges between 0.13% and 0.7% following cataract surgery [58,59,60] and between 0.03 and 0.13% after PPV [61]. The large diffusion of intravitreal injections has drastically increased the volume of intraocular procedures in the population, with the result of performing some of these outside the operating room setting [62]. Nevertheless, recent studies reported a relatively low rate of post-injection infections ranging between 0.02% and 0.082% [63,64,65,66,67,68].

Post-traumatic endophthalmitis accounts for 25–31% of cases and the reported incidence rate of endophthalmitis following open-globe injury varied from 0% to 16.5% with evidence of reduction over the past 70 years [69].

Depending on the causative agent, two main categories are recognized: bacterial and fungal endophthalmitis. The predominant organism causing the infection depends on the source (vegetable matter or retained intraocular foreign body), route of spread (post-surgery, trauma, delayed onset or hematogenous dissemination), geographic location, and patient characteristics [70]. Among exogenous endophthalmitis, Gentile and coauthors observed that 85.1% were due to gram-positive bacteria, 10.3% were due to gram-negative bacteria, and 4.6% were due to fungi. The most common bacterial pathogens isolated are Staphylococcus epidermidis (30.3%), different species of coagulase-negative Staphylococcus (9.1%), Streptococcus viridans (12.1%), Staphylococcus aureus (11.1%), Enterobacteriaceae (3.4%), and Pseudomonas aeruginosa (2.5%) [71].

## 6. Therapy

The key points in endophthalmitis treatment are infection control and eradication, inflammation management and re-infection prevention.

### 6.1. Medical Therapy

#### 6.1.1. Systemic Antibiotics

Systemic antibiotics in postoperative endophthalmitis are adopted as adjuvant therapy in some units despite little published evidence on their clinical efficacy [72]. As early as 1995, the EVS study concluded that a systemic treatment with ceftazidime 2 g every 8 h, amikacin 7.5 mg/kg initial dose followed by 6 mg/kg every 12 h did not affect the final visual outcome. Moreover, the authors hypothesized that the omission of systemic antibiotic therapy could reduce toxic effects, costs and length of hospital stay [1].

However, given the lack of univocal guidelines, adjunctive systemic antibiotic therapy is still commonly used resulting in various antibiotic regimens. The best-documented systemic drugs achieving therapeutic levels in vitreous appear to be fourth-generation fluoroquinolones [73], meropenem, and linezolid [74]. Conversely, patients with endogenous endophthalmitis seem to benefit from combined intravitreal and systemic antimicrobial therapy with lower rates of evisceration or enucleation [75].

#### 6.1.2. Topical Antibiotics

Broad-spectrum antibiotic combinations are usually started before a specific pathogen is identified and antibiotic sensitivity is determined. One of the drugs should be effective against Gram-positive organisms and the other one against Gram-negative organisms. Once antibiotic sensitivity is determined, targeted intervention should be undertaken. In the presence of corneal ulcer or wound abscess, fortified drops (including cefazolin 5%, tobramycin 1.4% and Vancomycin) should be used.

In the EVS study, the therapeutic regimen consisted of vancomycin (25 mg/0.5 mL) and ceftazidime (100 mg/0.5 mL) for subconjunctival injection, and topical vancomycin (50 mg/mL) and amikacin (20 mg/mL) every 4 hourly for routine cases or every hour if wound leak was present [1].

#### 6.1.3. Intravitreal Antibiotics

Intravitreal antibiotics are a mainstay for the management of endophthalmitis. The intravitreal route represents the only way the highest safe level of antibiotic can be rapidly delivered in the vitreous chamber.

An early injection is crucial because pathogens continue to replicate over time, produce toxins and dramatically damage the surrounding environment leading to irreversible visual loss. High doses of antibiotics are needed to keep their levels above the bacterial minimal inhibitory concentration as long as possible, given the variable speed of antibiotic clearance depending on the surgical and inflammatory status of the eye.

In vitrectomized eyes, if on one hand there is a higher risk of transient neurotoxicity due to macular pooling of the drug after injection, on the other hand, the absence of the vitreous body entails a quicker antibiotic clearance and drug concentration may sooner decrease to subtherapeutic levels [76].

As intravitreal injection usually takes place before or simultaneously to tap biopsy, broad-spectrum antibiotics are used to cover both gram-positive and gram-negative organisms. The most injected antibiotics are Vancomycin 1 mg/0.1 mL (for coverage of gram-positive organisms), Ceftazidime 2.25 mg/0.1 mL or Amikacin 0.4 mg/0.1 mL (for gram-negative organisms).

Amikacin seems to have a worse side-effect profile than Ceftazidime given the risk of macular infarction. Reddy and colleagues found similar bacterial resistance percentages for both antibiotics (18% for Ceftazidime and 13% for Amikacin) concluding that Ceftazidime still represented a first-line anti-gram-negative choice [77]. However, if nonresponse is observed, Amikacin could be an effective second-line choice.

### 6.2. Surgical Therapy

The EVS concluded that early vitrectomy in endophthalmitis was only beneficial to patients with vision of light perception or worse; however, this was a secondary finding as the study had not been designed for such subgroup analysis. Also, in EVS only a “core” vitrectomy was performed [78].

Already in 2005 Kuhn and Gini questioned the EVS indications on vitrectomy showing their results in a case series of 47 patients who had undergone early vitrectomy for endophthalmitis, with a ‘complete’ surgical vitrectomy as opposed to “core”: 91% of the cases achieved final acuity of 20/40 or better, as opposed to 53% in the EVS group [2]. The authors suggested that removing the posterior vitreous was advantageous in clearing the toxic load away from the macula whereas the EVS approach may have led to ‘macular hypopyon’, resulting in long-term dysfunction.

In recent years, with increased experience and surgical technique refinement, a more proactive stance in favour of vitrectomy has been adopted by many surgeons, outdating the EVS indications [72].

Consequently, new questions arose: Does SO tamponade after vitrectomy lead to better results? How does it affect microbial replication? Which patients would profit more from silicone oil tamponade?

The use of SO tamponade after completion (PPV) for endophthalmitis is debated. Several studies reported better visual outcomes in endophthalmitis patients treated with vitrectomy and SO tamponade, however other authors expressed concerns regarding the effectiveness and the true utility of this procedure.

## 7. Antimicrobial Activity of Silicon Oil

Among SO properties, antimicrobial activity has been extensively investigated. It has been suggested that the high surface tension and low permeability of SO could limit the freedom of movement of the pathogens, concentrating them in the ciliary body or close to the retinal blood vessels where the defence mechanisms could act more effectively [79]. Moreover, the space-occupying action of a long-standing tamponade may play an important role in pathogens’ and toxins’ wash-out, preventing the damage of the delicate retinal structures [17,80].

In 1999 Ozdamar et al. evaluated SO (1300 centistokes, medical grade, autoclave sterilized) in vitro antimicrobial activity against the most common endophthalmitis pathogenetic agents (*S. aureus*, *S. epidermidis*, *P. aeruginosa*, *C. albicans*, *Aspergillus* spp.) [9]. The authors observed that bacterial replication was significantly reduced in SO medium in comparison with a balanced salt solution. The authors explained their results both with a reduced nutrient concentration in SO and with a direct toxic activity exerted by the low molecular weight silicone oil particles. These findings have been confirmed and extended in the following years: in 2012 the antimicrobial activity of three different silicone oils—Arciolane 1300 cSt (Arcadophta, Toulouse, France), Arciolane 5500 cSt (Arcadophta, Toulouse, France) and OxaneR Hd (Bausch & Lomb Inc., Waterford, Ireland)—on different pathogens (*Staphylococcus aureus, Staphylococcus epidermidis, Enterococcus faecalis, Bacillus* sp., *Pseudomonas aeruginosa*, *Candida albicans* and *Aspergillus fumigatus*) was tested. The authors found that OxaneR Hd had the highest antibacterial and antimycotic activity and after two weeks Candida and Aspergillus growth was inhibited as well. The antimicrobial activity could therefore depend on SO chemical composition [14].

Bactericidal and antimycotic activity of 1000 cSt and 5000 cSt silicone oil against multi-resistant pathogens has been reported as well (*Methicillin-Resistant Staphylococcus Aureus*, *Staphylococcus epidermidis*, *Pseudomonas aeruginosa multiresistant*, *Klebsiella pneumoniae multiresistant, Escherichia coli*, *Candida albicans*, and *Aspergillus*). The time to exert bactericidal activity was different between the two oils (20 days for 1000 cSt oil and 30 days for 5000 cSt oil), whereas fungistatic activity was similar [15].

In contrast, Adams and colleagues, in 2013, explored the in vitro antimicrobial properties of a 1000 cSt SO (Óleo de Silicone^®^, Ophthalmos, São Paulo, Brazil) against *Pseudomonas aeruginosa*, *Escherichia coli*, *Staphylococcus aureus*, *Staphylococcus epidermidis*, *Candida albicans*, *Klebsiella pneumoniae* and *Streptococcus pneumoniae*; in all cases, no inhibition halo was observed [12]. An explanation for the different results of this study relies on the method: while other researchers used bacterial suspensions and observed the bacterial growth on different mediums in terms of Colony-forming unit, in the latter study, filter paper discs containing SO were placed in seeded plates and inhibition halo was observed. The Authors finally concluded that the SO at 1000 cSt did not have any effect on in vitro bacterial growth. They hypothesized that the substitution of the vitreous with SO could expose intraocular pathogens to nutritional deprivation leading them to death by starvation. This mechanism could explain the previously observed in vivo efficacy of silicone oil in the control of the infectious process [12].

The in vitro antimicrobial activity of SO against anaerobic agents, *specifically Propionibacterium acnes*, *Peptostreptococcus* spp., *Peptostreptococcus anaerobius*, *Bacteroides fragilis*, *Fusobacterium* spp., and *Clostridium tertium* has been investigated [10]. After a prolonged incubation of 7 days, 9.2 × 10^6^ colonies were observed in the silicone oil for Propionibacterium acnes, which may have been due to its biofilm formation capabilities. Additionally, Propionibacterium acnes produces propionic acid as a metabolic product, and the chemical effect of propionic acid on SO is not known. This chemical interaction may have contributed to the retention of bacterial viability in the SO [10].

Another evidence of biofilm-producing capabilities causing SO resistance has been reported in a case of endophthalmitis following retinal detachment surgery with SO injection at the end of the procedure. *M. abscessus*, a notorious organism, was isolated after gene sequencing; it tends to form a biofilm, hence becoming highly resistant to antibiotics. In this case, the antibiogram sensitivity chart showed sensitivity only to piperacillin-tazobactam [81].

Finally, SO seems to lack effectiveness against *Fusarium* spp., the most frequently isolated fungus after Aspergillum. In these cases, a central vitrectomy may be preferred to a complete one due to lower visibility and consequently higher risk of iatrogenic damage. SO tamponade is not recommended given the absence of proven fungicidal activity, moreover, antimycotic drug concentration may change in silicone oil [37,47].

In cases of fungal endophthalmitis associated with retinal detachment, it could be appropriate to perform retinal detachment surgery as soon as the infection resolves [82].

## 8. Anatomical and Visual Outcomes

Does substituting the vitreous humor with SO affect anatomical and visual outcomes?

Back in 2009, SO injection after complete PPV proved to lead to earlier infection control, better anatomical and visual outcomes, and a lower re-intervention rate in comparison with only intravitreal antibiotic injection [49]. In 2012 Patel et al. confronted the results obtained in 129 endophthalmitis patients treated with and without SO injection PPV in a prospective randomized clinical trial. The use of SO was related to better anatomical outcomes and a reduced number of reinterventions, the latter related to better visual outcomes. These results were even better in the post-traumatic endophthalmitis subgroup. The authors explained that in the silicone oil group, complete PPV with a surgically induced posterior vitreous detachment and a thorough examination of the peripheral retina led to overall better visual improvement. SO also prevents retinal detachments due to missed peripheral retinal breaks [53]. The results are in line with previous studies assessing the overall enhanced outcomes in patients treated with silicone oil injection [31,32,44,48,49,55].

The protective action exerted by SO has been confirmed in eyes at high risk for infections: the 2-year cumulative incidence of endophthalmitis was 31.2% in patients who received Boston Type 1 keratoprosthesis alone versus 0% in the group who received PPV and SO injection [83].

## 9. Retinal Detachment and Endophthalmitis

Retinal detachment is a serious condition that can dramatically undermine a patient’s visual prognosis; its occurrence in the context of endophthalmitis (concurrent or delayed onset) has variable incidence rates reported in literature ranging from 4.6% to 16% [29,84,85,86,87].

A retrospective case series of patients suffering from endophthalmitis, and retinal detachment treated with PPV and SO injection analyzed anatomical and visual outcomes among two groups. Group 1 included patients with concurrent endophthalmitis and retinal detachment, Group 2 included patients with delayed onset retinal detachment. The retinal reattachment rate was higher in the delayed onset group; however, the final visual outcome did not show statistically significant differences. The authors concluded that SO was effective in the management of retinal detachment related to endophthalmitis although low visual outcomes are likely to be expected [29].

Farouk and colleagues observed better infection control and lower postoperative retinal detachment rates in post-cataract endophthalmitis patients treated with PPV and SO injection [34]. Although postoperative visual acuity was not significantly better in eyes treated with SO injection, in a subgroup analysis, the number of patients with worsened visual acuity after the intervention was lower in the SO group. The authors, therefore, concluded that SO may play a role in preventing visual deterioration [34].

Similarly, Lin and colleagues observed lower postoperative retinal detachment rates in eyes with endophthalmitis treated with PPV and SO injection [52].

## 10. Traumatic Endophthalmitis

Several studies observed better anatomical and functional results after SO tamponade in patients with endophthalmitis following eye trauma; the explanations given have been multiple.

A complete vitrectomy (and not just a core vitrectomy) is needed to inject SO; this may help to remove more pathogens and to prevent post-operative vitreoretinal proliferation. When a complete vitrectomy cannot be performed because of low visibility, residual pathogens may indeed cause proliferative damage and hinder visual recovery [39].

Moreover, the use of SO creates compartmentalization of the eye allowing systemic antibiotics to concentrate between the retina and the oil bubble. Finally, SO prevents postoperative hypotony and ciliary body shock [44].

In 2019, a large study involving 98 eyes with post-traumatic endophthalmitis observed that low preoperative visual acuity, intraocular foreign bodies, and repeated intravitreal antibiotic injections were associated with worse visual outcomes. Patients who underwent SO tamponade received fewer intravitreal injections, ending up with better visual results [56].

Zhou and colleagues, on the other hand, observed worse results in patients who received primary SO tamponade compared with those who received Octafluoropropane (C3F8); these results, however, may be biased by the worse condition at presentation in the SO group [51].

Finally, in cases of traumatic endophthalmitis with an intraocular foreign body, different approaches have been proposed, according to the grade of retinal damage. If no retinal damage was intraoperatively detected, a balanced salt solution could be the tamponade of choice, reserving gas tamponade to cases with limited retinal damage and employing SO in eyes with large retinal breaks or proliferative disease. This strategy could spare unnecessary procedures and could be more cost-effective [38,50].

## 11. Endogenous Endophthalmitis

In 2014, Do and colleagues conducted a Vietnamese randomized clinical trial to compare two different treatment pathways in 108 patients with severe bacterial endogenous endophthalmitis [45]. A first group was treated with intravitreal antibiotic injections + PPV and a second group with intravitreal antibiotic injection + PPV + SO tamponade.

Nine months after surgery the second group showed better visual acuity and overall better results (anatomical and reintervention rates); therefore, according to the authors, in cases of severe bacterial endophthalmitis, PPV should be followed by SO injection.

## 12. Endophthalmitis in Silicone Oil Filled Eyes

There are few reported cases of endophthalmitis after SO injection, the causative pathogens being *Pseudomonas Aeruginosa* [21,88,89], coagulase-negative *Staphilococcus* [90], *S. pneumoniae* [89] and *Mucorales* spp. *Mucormycosi* [91].

In most cases, the chosen approach was to remove SO, inject antibiotics in the vitreous chamber and then fill the eye again with SO. In a case of *Mucorales* spp. endophthalmitis, the patient refused further surgical procedures leading to phthisis bulbi and eye loss [91].

Steinmetz and colleagues describe their experience in two cases of endophthalmitis in SO-filled eyes after vitrectomy treated with an antibiotic injection directly in a SO-filled vitreous cavity [27]. Only in one patient anterior chamber puncture was performed with negative bacterial and leukocyte counts. In one case half of the usual antibiotic (vancomycin and Ceftazidime) dose was used whereas in the other full dose was administered; in neither case, retinal toxicity was observed, and the infection was resolved in all cases.

The antibiotic might have injected occupied the space between silicone oil and the retina where bacteria are more likely to replicate. This approach could be advantageous in eyes that cannot be operated on straight away and spare additional surgeries in multi-operative eyes.

## 13. Limitation of Silicone Oil Use

Performing a complete vitrectomy before SO injection is not always possible in eyes with severe endophthalmitis; firm adherence of inflammatory debris to the retina and fundus visualization problems due to corneal oedema and pupil membranes may complicate the surgical procedures. Moreover, the use of SO during the surgical treatment of endophthalmitis has some disadvantages, including the retinotoxic potential and the observation that not all pathogens are inhibited by this substance.

SO low-molecular weight compounds may diffuse out of the vitreous cavity, resulting in retinal toxicity and affecting eye physiology by absorption of lipophilic molecules from the ocular environment [92].

It has been suggested that substances like cellular debris, inflammatory substances and blood products may enhance early emulsification [31].

Vitreous humor plays a crucial role in eye physiology and must not be considered an inert optical medium; it is central in intravitreal pharmacodynamics and its buffering capacities have been demonstrated [93].

Replacement of vitreous humor with SO may interfere with toxin washout. Several defence mechanisms within the retina such as the blood-retinal barrier, cytochrome p450 activity and antioxidants protect the retina against a variety of insults. Since the waste products of the retina diffuse into the vitreous, the effective volume of the vitreous space is also important in detoxification as the amount of this space is inversely related to the concentration of toxic materials. Therefore, with the reduction of effective vitreous space, the tolerable number of acids or toxins is likely to be reduced. In the context of endophthalmitis with a high retinal metabolic rate, increased production of waste products, and acidification of the retinal environment, replacing vitreous with SO may expose the retina to more rapid decompensation and damage [94].

Dosing intravitreal antibiotics in SO-filled eyes can be challenging. The physician will struggle both with lower retinal toxicity thresholds and with a higher antibiotic clearance; the medication becomes more dangerous and stays in the vitreous chamber for less time [95].

SO causes compartmentalization in the eye that may trap inflammatory debris between the SO and retina, leading to the development of pre-retinal membranes that could cause retinal tears. The presence of SO and consequent compartmentalization may also influence the distributions of intravitreal antibiotics that can reach toxic concentrations. In addition, the toxicity of SO for the optic disc should be seriously considered [96].

Many authors, therefore, concluded that SO following PPV for endophthalmitis should be reserved only for cases with trauma history and/or associated retinal detachment.

## 14. Discussion

In this review, we examined 44 articles aiming to answer the main questions that we considered for this work. Firstly, we focused on the antimicrobial activity of SO and its contribution to controlling the infection (Table 1). 5 out of 7 in vitro experimental studies demonstrated antimicrobial activity against different species of bacteria (e.g., *S. aureus, S. epidermidids, P. aeruginosa, C. albicans*) and fungi (*Asperigillus* spp., *C. albicans*) [9,11,13,14,15]. They employed different types of SO, observing a greater antimicrobial effect with heavy SOs. In contrast, 2 studies [10,12] did not observe inhibition on growth against the species examined (e.g., *Propionibacterium acnes, Pseudomonas aeruginosa; Escherichia coli; Staphylococcus aureus; Staphylococcus epidermidis; Candida albicans; Klebsiella pneumoniae; Streptococcus pneumoniae*). A possible explanation for the different results among studies could rely on different methodologies to assess antimicrobial activity. Some authors inoculated bacterial suspensions on different SO mediums; others used filter paper discs containing SO, placed in seeded plates. In addition, we found 3 case series [26,27,28] and one case report [21] of postoperative endophthalmitis in SO-filled eyes.

The use of SO as endotamponade associated with PPV lead to better outcomes and provided better control of the infection in all 4 randomized clinical trials, in 15 out of 18 case series and in 3 out 5 retrospective interventional studies. In 3 case series [38,41,42] and 2 retrospective studies [51,56], there was no difference in visual acuity between eyes that received SO tamponade and controls (Table 2). However, in some studies, patients that received SO had worst visual acuity and more severe ocular damage compared with eyes that received other tamponades. The use of SO contributed to a better visual and anatomical outcome, reduced the need for additional surgery [30,32,48,49,53], decreased the risk of retinal detachment [29,30,32,49] and was considered an eligible option for intravitreal injection of antibiotics [35]. Early PPV with endotamponade should be preferred to PPV without tamponade [46,55]. Some Authors reserved PPV and endotamponade only in complicated cases associated with corneal involvement, fungal endophthalmitis and cases requiring intraocular lens removal [36].

A limitation of this review was related to the typology of the studies included since most of them were case reports and case series that implicated a lack of a control group. Furthermore, we excluded editorials, letters to editors and articles that were not in English.

In conclusion, injections of SO after PPV in cases of endophthalmitis may entail several advantages. SO reduces the risk of postoperative retinal detachment, especially in case of undetected retinal breaks, produces compartmentalization of the eye, may lead to early visual recovery due to an optically clear medium, allows laser photocoagulation in case of missed retinal breaks, prevents the occurrence of severe hypotony in the early postoperative period and has antimicrobic activity due to an inhibitory effect for several species of pathogens. However, concerns regarding possible toxic effects on the retina and optic disc, compartmentalization and impaired washout of pathogen toxins have been reported. SO may also influence intravitreal antibiotic distribution and clearance.

## Figures and Tables

**Table 1:** Does silicone oil have antimicrobial activity?

Author	Year	Study Design	Silicone Oil	Purpose	Pathogens Studied	Outcomes	Antimicrobial Activity
Ozdamar et al. [9]	1999	In vitro experimental study	1300 cSt	Compared Microorganism growth in saline, growth medium and silicone oil medium to analyze SO antimicrobial activity	*S. aureus*, *S. epidermidids*, *P. aeruginosa*, *C. albicans* and *Asperigillus* spp.	CFU number	**YES** (Silicone oil showed antimicrobial activity against *S. aureus*, *S. epidermidids*, *P. aeruginosa*, *C. albicans* and *Asperigillus* spp.)
Arici et al. [10]	2016	In vitro experimental study	1300 cSt	To investigate the in vitro antimicrobial activity of silicone oil against anaerobic agents	*Propionibacterium acnes*, *Peptostreptococcus* spp., *Peptostreptococcus anaerobius*, *Bacteroides fragilis*, *Fusobacterium* spp., and *Clostridium tertium*	CFU number	**NO** (Propionibacterium acnes showed resistance against the antimicrobial effect of silicone oil)
Economou-Stamatelopoulou et al. [11]	2004	In vitro experimental study	1000 cS and 5000 cSt	To investigate the in vitro antifungal activity of silicone oil	*Aspergillus* spp.	CFU number	**YES** (SO and PFCL conform to standards, effective against *Asperigillus* spp.)
Adams et al. [12]	2012	in vitro experimental study	1000 cSt	To verify the effect of the silicon oil on in vitro bacterial growth of selected microorganisms.	*Pseudomonas aeruginosa*; *Escherichia coli*; *Staphylococcus aureus*; *Staphylococcus epidermidis*; *Candida albicans*; *Klebsiella pneumoniae*; *Streptococcus pneumoniae*	Inhibition halos, CFU	**NO** (The silicon oil 1000 cps does not have any effect on the bacterial growth of any of the studied microorganisms. *Pseudomonas aeruginosa*; *Escherichia coli*; *Staphylococcus aureus*; *Staphylococcus epidermidis*; *Candida albicans*; *Klebsiella pneumoniae*; *Streptococcus pneumoniae*)
Ornek et al. [13]	2014	In vitro experimental study	conventional silicone oil (RS OIL 5000) and heavy silicone oil (heavy Sil 1500)	To compare the effectiveness of conventional silicone oil and heavy silicone oil against endophthalmitis-causing agents	*S. aureus*, *S.epidermidis*, *E. coli*, *P. aeruginosa*, and *C. albican*	CFU number	**YES** (Conventional silicone oil decreased the colony numbers of all bacteria except for *C. albicans*, but heavy silicone oil demonstrated a superior antimicrobial effect on all pathogens including *C. albicans*)
Chrapek et al. [14]	2012	In vitro experimental study	Arciolane 1300 centistokes, Arciolane 5500 centistokes and Oxane Hd, heavy silicone oil	To investigate and compare the antimicrobial activity of three types of silicone oils used in ophthalmic surgery (Arciolane 1300 centistokes, Arciolane 5500 centistokes and Oxane Hd, heavy silicone oil)	*Staphylococcus aureus*, *Staphylococcus epidermidis*, *Enterococcus faecalis*, *Bacillus* sp., *Pseudomonas aeruginosa*, *Candida albicans* and *Aspergillus fumigatus*	CFU number	**YES** (The Oxane Hd silicone oil exhibited the highest antimicrobial activity, both antibacterial and antifungal. It inhibited the growth activity of all inoculated bacteria, albeit after various times; after 14 days, it acted upon candida and aspergilli as well)
Dave et al. [15]	2019	In vitro experimental study	Aurosil 1000 cSt, Aurosil Plus 5000 cSt	To test the antimicrobial properties of silicon oil (Aurosil 1000 cst, Aurosil Plus 5000 cst) on in vitro growth of common microorganisms causing endophthalmitis	*Staphylococcus aureus*, *Staphylococcus epidermidis*, *Pseudomonas aeruginosa*, MDR strain of *Klebsiella pneumoniae*, *Escherichia coli*, *Candida albicans*, and *Aspergillus flavus*	CFU number	**YES** (For 1000 cSt SO, complete bactericidal action was noted by day 21 of exposure, and for the 5000 cSt SO, it was noted by day 30. Both the oils show a similar fungistatic action)
Okonkwo et al. [26]	2018	Case series	5000 cSt	To report the long-term outcome of the management of a series of culture-proven post pars plana vitrectomy endophthalmitis in which the infective agent was in the silicone oil used as an endotamponade	*Burkholderia cepacia*	BCVA e and Anatomical outcomes	**NO** (Gram-negative bacilli can colonize silicone oil resulting in post pars plana vitrectomy endophthalmitis)
Steinmetz et al. [27]	2018	Case series	5000 cSt	to describe 2 cases of endophthalmitis successfully treated with an office injection of intravitreal antibiotics.	Not Applicable	BCVA, IOP, Anatomical outcomes	**NO** **Infectious endophthalmitis in silicone oil-filled eyes**.
Tayyib et al. [28]	1997	Case series	Not reported	To assess the onset of sterile endophthalmitis in silicone oil-filled eyes	Not Applicable	Postoperative complication rate, incidence of endophthalmitis after vitrectomy and silicone oil tamponade	**YES** (Silicone oil showed antimicrobial activity against *S. Aureus*, *S. Epidermidids*, *P. aeruginosa*, *C. albicans* and *Asperigillus* spp.)
Goel et al. [21]	2015	Case report	1000 cSt	to report the first case of multidrug-resistant endophthalmitis following pars plana vitrectomy in a silicone oil-filled eye	*Pseudomonas aeruginosa*	At 1 month there was an attached retina and resolved periphlebitis	**NO** endophthalmitis in silicone filled eyes (Pseudopmonas). Silicone oil may be an impediment to intravitreal delivery of antibiotics, as it may result in dangerously high concentrations.

BCVA: Best Corrected Visual Acuity; cSt: Centi Stokes; CFU: colony-forming unit; SO: Silicone Oil; IOP: Intraocular Pressure; MDR: multidrug-resistant.

**Table 2:** Does Silicone oil injection after vitrectomy improve visual and anatomical outcomes?

Author	Year	Study Design	Sample	Groups	Purpose	Anatomical Outcomes	Visual Outcomes
Dave et al. [29]	2017	Case Series	93	Group 1 (concurrent RD), Group 2 (Delayed RD).	To analyze the treatment outcomes in patients with endophthalmitis and concurrent or delayed-onset retinal detachment managed with pars plana vitrectomy, intravitreal antibiotics, and silicone oil.	The complete retinal reattachment rate was 73.7% in Group 1 and 98.5% in Group 2.	Visual acuity of 20/400 or better rate was 30.0% in Group 1 and 39.7% in Group 2
Hudieb et al. [30]	2012	Case series	34	Groups 1, 2 and 3 were treated initially without silicone oil, Group 4 was treated initially with silicone oil.	To evaluate the role and outcomes of PPV and oil injection in the treatment of infectious endophthalmitis.	In 22 patients (groups 1, 2 and 3) (55%) who needed further surgery, either for persistent infection or retinal detachment 12 patients (group 4) treated at first with silicone oil had a rapid control of the infectious process and better anatomical results with this procedure only.	Final visual acuity was better in the silicone oil groups (groups 3 and 4) than in the non-silicone groups (groups 1 and 2).
Olgun et al. [16]	2018	Case Report	2	none	To report cases of Endophthalmitis after Xen Gel stent implantation treated with PPV and SOI	Infection control and attached retina were obtained in both cases.	Final visual acuity was hand motion in Case 1 and 20/400 in Case 2
Baxter et al. [17]	2015	Case Report	1	none	To report a case of S. Mitis endophthalmitis treated first with PPV and IOAB, then SOI.	At postoperative Month 1 attached retina with improved retinal perfusion on fluorescein angiography was observed.	At postoperative Month 1 BCVA was 20/200
Aras et al. [31]	2001	Case Series	6	none	To investigate the use of silicone oil in patients who had undergone vitrectomy for the treatment of endophthalmitis associated with retinal detachment	Final retinal reattachment and treatment of endophthalmitis were achieved in 5 eyes at the end of follow-up	Final visual acuity was 20/40 in 1 case, counting fingers in 4 cases and no light perception in 1 case.
Bali et al. [32]	2003	Case Series	34	(group 1), PPV + IOAB. (group 2) PPV + IOAB; PPV (group 3) PPV + IOAB; PPV + IOAB + SOI. (group 4), PPV + IOAB + SOI	To evaluate the role of PPV and silicone oil injection in the treatment strategy of severe endophthalmitis.	In 22 patients (groups 1, 2 and 3) (55%) who needed further surgery, either for persistent infection or retinal detachment 12 patients (group 4) treated at first with silicone oil had a rapid control of the infectious process and better anatomical results with this procedure only.	Final visual acuity was better in the silicone oil groups (groups 3 and 4) than in the non-silicone groups (groups 1 and 2).
Pinarci et al. [33]	2013	Case Series	8	none	To report the role of early vitrectomy and silicone oil tamponade in acute endophthalmitis following intravitreal injection.	There was no retinal detachment or phthisis bulbi during the follow-up period (1–4 years).	BCVA at final follow-up was 0.05 in two patients (25%), 0.1 in three patients (37.5%), 0.3 in two patients (25%), and 0.8 in one patient (12%).
Farouk et al. [34]	2017	Case Series	26	Group 1 in which vitrectomy was done without silicon oil and group 2 in which vitrectomy was done with silicon oil. All cases were followed for 6 months.	To evaluate the outcomes of vitrectomy with and without silicone oil injection for the treatment of infectious endophthalmitis after cataract surgery when the retina is severely affected.	Four cases (19%) suffered from the persistence of infection after vitrectomy and 3 cases (14.2%) had a postoperative retinal detachment in group 1. These complications were not reported in any case of group 2.	In Group 1 the mean logMAR visual acuity was 2.09 ± 0.82, in Group 2 the mean logMAR visual acuity was 2.04 ± 0.75.
Yan et al. [35]	2008	Case Series	18	none	To explore the effects of vitrectomy combined with silicone oil injection in the treatment of traumatic endophthalmitis without retinal detachment and analyze the relative factors.	There was no retinal detachment or ocular atrophy.	The postoperative visual acuity ranged from light perception to 0.8. The visual acuity increased in 15 eyes (83%) and was stable in 3 eyes (17%).
Lin et al. [52]	2011	Case Series	62	Patients were divided into groups according to the VA at presentation. Group A (12 cases; VA = LP) and group B (50 cases; VA > LP).	The authors investigated initial ocular conditions, surgical management and outcomes of PTE patients and analyzed their relationship to find the necessary management for different patients’ conditions.	Initial VA, preventive scleral buckling and silicone oil tamponade may be good predictors of anatomic outcome.	In conclusion, for PTE, 69.4% (43/62) of the eyes with a good final visual outcome (VA >/= HM) were successfully managed with PPV, and six of them underwent silicone oil injection.
Khaqan et al. [46]	2017	Prospective Study	112	In group 1 patients undergoing PPV with endotamponade (silicon oil) Group 2 patients undergoing PPV without endotamponade.	To evaluate the anatomical and functional outcomes of pars plana vitrectomy (PPV) in acute postoperative endophthalmitis with or without endotamponade.	23 (76.66%) patients out of 30 who underwent PPV only (Group 2) showed retinal detachment within the first four weeks of follow-up, while among 30 patients of Group 1 who underwent PPV with endotamponade, no patient showed retinal detachment in the first four weeks post-operatively.	Seventy-six (92.68%) participants showed improved vision (6/36-6/60) in Group 1 and in Group 2, 07 (23.33%) participants showed improved vision (6/36-6/60).
Mohd-Ilham et al. [18]	2019	Case Report	1	none	To report a case of a large subretinal abscess secondary to Klebsiella pneumoniae endophthalmitis in a pyelonephritis patient.	The patient showed complete regression of the intraocular inflammation and subretinal abscess.	The patient regained her vision on 6/36.
Mackiewicz et al. [24]	2000	Case Report	1	none	the patient was treated with three vitrectomies. During the third vitrectomy, the retinal detachment was repaired with a circumferential buckle and silicone oil tamponade.	If inflammatory changes of the retina are found during surgery, it seems advisable to administer silicone oil as a protection against a detachment of the retina.	During the 2-year observation period, the visual acuity in the present case was 0.1.
Kapoor et al. [55]	2012	Interventional consecutive retrospective study	30	Group 1 (*n* = 14) PPV + 1000 centistoke silicone oil tamponade for 12 weeks; Group 2 (*n* = 16) PPV + 1000 centistoke silicone oil tamponade for 24 weeks.	to evaluate the efficacy of early vitrectomy with adjunctive silicone oil to treat endophthalmitis.	Additional surgery was required in 3% (1/30) of the study group.	At 9 months, 73% of all patients (22/30) achieved the best corrected visual acuity (BCVA) of 20/40 or better.
Kaynak et al. [48]	2003	Retrospective Study	56	Group 1 (*n* = 24) eyes core vitrectomy; Group 2 (*n* = 28) eyes total PPV, encircling band, silicone tamponade, and endolaser.	To evaluate the results of 2 surgical techniques in eyes with postoperative endophthalmitis.	The number of additional procedures was significantly less, and the rate of surgical success was significantly higher in Group 2 than in Group 1 (*p* < 0.01).	There was no statistically significant difference between the 2 groups in final visual acuity (*p* > 0.05).
Verma et al. [36]	2017	Case Series	9	none	Describes nine different real-world scenarios of endophthalmitis responding to intravitreal antibiotics alone and cases requiring intraocular lens removal, radical vitrectomy with hyaloid peeling, base dissection, and silicone oil	Not reported for all patients	Not reported for all patients
Krėpštė et al. [19]	2013	Case Report	1	none	To present a case of meningitis with bilateral endogenous bacterial panophthalmitis in a previously healthy individual.	Right eye was enucleated, two weeks after the removal of silicone oil the left eye suffered hypotony and subsequent phthisis bulbi.	At 13 weeks (BCVA) of the left eye was 0.07, Two weeks after the removal of silicone oil, visual acuity decreased to light perception.
Nagpal et al. [43]	2012	Prospective Randomized Study	129	Group 1 (*n* = 65) eyes, which underwent vitrectomy alone, were compared with group 2 (*n* = 64) eyes, in whom complete PPV with SOI was done.	To compare outcomes of pars plana vitrectomy (PPV) with and without silicone oil injection (SOI) in the surgical management of endophthalmitis.	Rate of retinal detachment was 6.2% in group 2 as compared with 25.5% in group 1. Groups 1 and 2 required additional subsequent procedures in 27 eyes (41.54%) and 5 eyes (7.8%), respectively (*p* < 0.0001).	Mean best corrected visual acuity improvement was 0.867 ± 1.13 and 1.140 ± 0.88 in groups 1 and 2, respectively (*p* < 0.005). In the posttraumatic subgroup, the difference between groups 1 and 2 in mean change in best corrected visual acuity was statistically significant (0.580 ± 1.10 and 1.132 ± 0.8 respectively, *p* < 0.05).
Cakir et al. [37]	2009	Case Series	8	none	to review microbiologic and medical records of eight cases of endophthalmitis caused by Fusarium species after cataract surgery.	All patients underwent multiple vitrectomies with silicone oil injections. One patient with corneal involvement underwent evisceration despite a variety of treatments. One patient with unregulated diabetes was pre phthisical without recurrence of infection. The final visual acuity of patients was between light perception and 20/100.	The final visual acuity of patients was between light perception and 20/100.
Azad et al. [44]	2003	Prospective Randomized Controlled Study	24	Group 1: core vitrectomy Group 2: complete vitrectomy with silicone oil endotamponade	to compare core vitrectomy with complete vitrectomy and silicone oil in posttraumatic endophthalmitis.	Four (33.33%) of the 12 patients in group 1 developed retinal detachment following vitrectomy. In group 2 Complete vitrectomy ensures the complete removal of vitreous membranes and prevents fibrous proliferation and tractional detachment.	In group 1, 41.66% of patients (5/12) achieved a useful visual outcome (≥20/400) and only one patient achieved a final visual acuity ≥20/200. In group 2, 75% of patients (9/12) had a visual outcome better than 20/400 (*p* = 0.07) and 58.3% (7/12) achieved a visual acuity greater than 20/200 (*p* = 0.02).
Siqueira et al. [49]	2009	Retrospective Study	35	Group 1: intravitreal antibiotic injection, associated with topical and oral antibiotics Group 2: vitrectomy with intravitreal antibiotic injection and silicone oil injection	To evaluate the outcomes of pars plana vitrectomy and silicone oil injection for the treatment of infectious endophthalmitis	Group 1: Six patients (25%) had retinal detachment during the first month of follow-up and also required PPV and SOI. Group 2: 2 patients (*n* = 11), all of them had controlled infection on the first procedure. In one case (9.09%), a severe proliferative vitreoretinopathy (PVR) induced loss of vision (NLP).	Group 1: Nine patients (37.5%) had worsening visual acuity, 10 patients (41.6%) improved and 5 patients (20.83%) did not change. Group 2: One patient (9.09%) showed worsening visual acuity, 5 patients (45.45%) improved and 5 patients (45.45%) did not change.
Wang et al. [50]	2011	Retrospective Study	36	Group 1: 4 eyes without obvious retinal damage with BSS. Group 2: Sixteen eyes that had mild retinal damage filled with C3F8 gas. Group 3: 16 eyes with serious retinal damage were treated with silicone oil.	To study the criterion-reference of endotamponade in pars plana vitrectomy for the metallic intraocular foreign body (MIOFD) associated with endophthalmitis	Group 1: There was no postoperative complication. Group 2: Only 2 cases occurred with postoperative retinal detachment. Group 3: higher incidence of postoperative complications (18.8% retinal detachment, 25% ocular Hypertension, 31.3% needed secondary surgical treatment).	Group1: The visual acuity (VA) was improved Group 2: postoperative VA improved in 10 eyes (62.5%), 4 eyes (25.0%) remained unchanged and 2 eyes (12.5%) decreased. Group 3: Postoperative VA of 9 eyes (56.3%) improved, 3 eyes (18.8%) remained unchanged and 4 eyes (25.0%) decreased.
Do et al. [45]	2014	Randomized Controlled Trial	108	Group 1 (53) standard PPV + IVT antibiotics. Group 2 (55) standard PPV + IVT antibiotics + silicone oil.	to compare treatment outcomes with and without silicone oil tamponade in patients undergoing (PPV for severe BEE.	The anatomical result in Group 2 also had a trend of being better (Group 1, 64.2% versus Group 2, 80%; *p* = 0.07).	The rate of VA improvement ≥ over baseline tended to be better in Group 2, 40% versus 22.6% in Group 1 (*p* = 0.0521).
Jiang et al. [38]	2017	Case Series	121	none	To evaluate visual outcomes and identify prognostic factors after PPV surgery for traumatic endophthalmitis.	none	The use of silicone oil tamponade was not a significant factor resulting in better BCVA.
Jin et al. [39]	2017	Case Series	107	none	to determine visual and anatomical outcomes of pediatric patients with posttraumatic endophthalmitis following 23 gauge PPV combined with silicone oil endotamponade	Anatomical recovery 91.59% Hypotony 0% Silicone oil sustained eyes 1.87% Silicone oil, low IOP 2.8% Atrophy 0.93% Uncontrolled inflammation 1.87% Evisceration in subsequent procedure 2.8% Evisceration in primary procedure 0%.	BCVAs were not only favourable, but also often better than those predicted by OTS.
Lu et al. [56]	2019	Retrospective Study	98	Group 1: only IV antibiotics (38). Group 2: IV antibiotics + PPV (30). Group 3: IV antibiotics + PPV + SO (27). Group 4: intracameral antibiotics (3).	to evaluate the prognostic factors associated with visual outcomes in the salvageable eyes with post-traumatic endophthalmitis between 2008 and 2015.	The silicone oil group had fewer repeated intravitreal injections than the group without oil tamponade.	The number of intravitreal injections was independently associated with poor visual outcomes.
Won et al. [25]	2013	Case Report	1	none	to report a case of acute postoperative endophthalmitis caused by vancomycin-resistant Staphylococcus hominis, treated with intraocular lens removal, and silicone oil tamponade.	At 3 months, the retina was attached.	At 3 months, the visual acuity of the silicone oil-treated eye was 20/400.
Siu et al. [22]	2015	Case Report	1	none	to report a case of endogenous endophthalmitis from *K. pneumoniae.*	At 3 months retinal re-detachment and reoperation.	At three months after the second operation BCVA 6/60.
Zhang et al. [40]	2015	Case Series	21	none	to evaluate the surgical efficacy and timing of 23-G vitrectomy for acute endophthalmitis following cataract surgery, and to determine when silicone oil tamponade and intraocular lens (IOL) removal are indicated during vitrectomy for endophthalmitis.	In all patients, the surgery resolved the endophthalmitis, one patient experienced a recurrence of endophthalmitis 2 months after vitrectomy. Two patients required primary silicone oil tamponade. In 2 other patients, retinal detachment occurred with subsequent vitrectomy and silicone oil tamponade.	Two patients (9.5%) had BCVA > 0.05 before treatment, 14 patients (66.7%) overall had BCVA > 0.05 after treatment. The difference was significant (χ^2^ = 15.003, *p* = 0.002).
Chon et al. [23]	2017	Case Report	1	none	To report successful management of late-onset Streptococcus mitis endophthalmitis treated by vitrectomy, panretinal photocoagulation (PRP) and silicone oil tamponade	One month after the surgery, intraocular inflammation was stabilized.	visual acuity was improved from light perception to 20/200.
Yospaiboon et al. [41]	2018	Case Series	417	none	To determine factors affecting visual outcomes after treatment of infectious endophthalmitis. Methods of treatment were medical treatment (18.71%) and surgical treatment (81.29%), including pars plana vitrectomy with or without silicone oil tamponade (62.59%) and destructive surgery (18.71%).	none	After treatment, visual improvement was noted in 44.6%, stable vision in 18.47%, and worse vision in 36.93%. Factors associated with improved visual outcomes were types of endophthalmitis, causative organisms, and initial visual acuity before treatment.
Yospaiboon et al. [42]	2018	Case Series	45	none	To evaluate visual outcomes and possible predictive factors in the treatment of infectious endophthalmitis caused by *Streptococcus* species. Methods of treatment were medical treatment (18.71%) and surgical treatment (81.29%), including pars plana vitrectomy with or without silicone oil tamponade (62.59%) and destructive surgery (18.71%).	none	Nine patients (20%) had improved vision after treatment. The only predictive factor associated with improved visual outcomes was vitrectomy within 3 days. Medical treatment and PPV with antibiotics demonstrated more improved visual outcomes than PPV with antibiotics and silicone oil tamponade, but the difference was not statistically significant (*p* = 0.072).
Zhou et al. [51]	2020	Retrospective Study	22	Primary PPV + SO:18 eyes. Primary PPV + C3F8: 4 eyes.	To explore the traumatic endophthalmitis in young children and the outcomes of pars plana vitrectomy	Five patients had retinal detachment (RD) within 3–4 d of initial presentation. Four patients had traction RD after the second PPV, as a complication of surgery. Four patients exhibited band-shaped degeneration of the cornea during follow-up after the third operation. The final IOP was 8.9 ± 1.8 mm Hg.	The final BCVAs were 20/200 or better in five patients, two patients could count fingers, eight patients could detect hand movement, one patient had light perception and one patient had no light perception. Final BCVAs were not available for three patients. Whose (66.7%) had retinal injury exhibited worse BCVA (*p* = 0.019, Fisher’s exact test). Eyes underwent SO tamponade exhibited worse final BCVA than that with C3F8 in the primary PPV (*p* = 0.026, Fisher’s exact test).

BSS = balanced salt solution; RD: Retinal Detachment; PPV: Pars Plana Vitrectomy; SO: Silicone Oil; SOI: Silicone Oil Injection; VA: Visual Acuity; BCVA: Best Corrected Visual Acuity; IOAB: Intraocular Antibiotic; IOP: Intraocular Pressure; MIOFD (Metallic Intraocular Foreign Bodies; HM: Hand Motion; IVT: Intravitreal Injection Therapy; BEE (endogenous bacterial endophthalmitis); OTS: Ocular Trauma Scores. The level and the strength of evidence were defined according to the Oxford Centre for Evidence-Based Medicine (OCEM) 2011 guidelines and the Scottish Intercollegiate Guideline Network (SIGN) assessment system for individual studies as implemented for Preferred Practice Patterns by the American Academy of Ophthalmology respectively [14,15]. The quality of evidence based on the Grading of Recommendations Assessment, Development and Evaluation (GRADE) system was also assessed [16].

## Data Availability

All data supporting this work are included in the manuscript and/or Supplementary Materials. The protocol was prepared and can be requested from the authors.

## Supplementary Materials

The following supporting information can be downloaded at: https://www.mdpi.com/article/10.3390/jcm11185445/s1, Table S1: Does silicone oil have antimicrobial activity? The quality of evidence based on the Grading of Recommendations Assessment, Development and Evaluation (GRADE) system. Table S2: Does Silicone oil injection after vitrectomy improve visual and anatomical outcomes? The quality of evidence based on the Grading of Recommendations Assessment, Development and Evaluation (GRADE) system. Table S3: Risk of Bias (ROB) according to Revised Cochrane risk-of-bias tool for randomized clinical trials. Table S4: A detailed Newcastle-Ottawa Scale of each included retrospective study. Table S5: Joanna Briggs Institute (JBI) Critical Appraisal Checklist for Case series. Table S6: Joanna Briggs Institute (JBI) Critical Appraisal Checklist for Case Repots. File S1: PRISMA 2020 checklist.
