# Supplementary material for: The Role of Silicone Oil in the Surgical Management of Endophthalmitis: A Systematic Review
Source: J Clin Med. 2022 Sep 16;11(18):5445. doi: 10.3390/jcm11185445 (PMC9505397; doi:10.3390/jcm11185445)
Supplement: Supplementary file 1 [file jcm-11-05445-s001.zip › jcm-1849201-supplementary-Table S1.pdf]

Table S1. Does silicone oil have antimicrobial activity?

Author	Year	Study Design	Silicone Oil	Purpose	pathogens studied	Outcomes	Level	Strength	Grade
Ozdamar et al.[9]	1999	In vitro experimental study	1300 cSt	Compared Microorganism growth in saline, growth medium and silicone oil medium to analyze SO antimicrobial activity	S.Aureus, S.Epidermidids, P. aeruginosa, C.albicans and Asperigillus spp.	CFU number	5	III	Very low
Arici e t al. [10]	2016	In vitro experimental study	1300 cSt	To investigate the in vitro antimicrobial activity of silicone oil against anaerobic agents	Propionibacterium acnes, Peptostreptococcus spp., Peptostreptococcus anaerobius, Bacteroides fragilis, Fusobacterium spp., and Clostridium tertium	CFU number	5	III	Very low
Economou-Stamatelopoulou et al. [11]	2004	In vitro experimental study	1000 cS and 5000 cSt	To investigate the in vitro antifungal activity of silicone oil	Aspergillus spp	CFU number	5	III	Very low
Adams et al. [12]	2012	in vitro experimental study	1000 cSt	To verify the effect of the silicon oil on in vitro bacterial growth of selected microorganisms.	Pseudomonas aeruginosa; Escherichia coli; Staphylococcus aureus; Staphylococcus epidermidis; Candida albicans; Klebsiella pneumoniae; Streptococcus pneumoniae	Inhibition halos, CFU	5	III	Very low
Ornek et al. [13]	2014	In vitro experimental study	conventional silicone oil (RS OIL 5000) and heavy silicone oil (heavySil 1500)	To comparing the effectiveness of conventional silicone oil and heavy silicone oil against endophthalmitis-causing agents	S. aureus, S.epidermidis, E. coli, P. aeruginosa, and C. albican	CFU number	5	III	Very low
Chrapek et al. [14]	2012	In vitro experimental study	Arciolane 1300 centistokes, Arciolane 5500 centistokes and Oxane Hd, heavy silicone oil	To investigate and comparing the antimicrobial activity of three types of silicone oils used in ophthalmic surgery (Arciolane 1300 centistokes, Arciolane 5500 centistokes and Oxane Hd, heavy silicone oil)	Staphylococcus aureus, Staphylococcus epidermidis, Enterococcus faecalis, Bacillus sp., Pseudomonas aeruginosa, Candida albicans and Aspergillus fumigatus	CFU number	5	III	Very low
Dave et al. [15]	2019	In vitro experimental study	Aurosil 1000 cSt, Aurosil Plus 5000 cSt	To test the antimicrobial properties of silicon oil (Aurosil 1000 cst, Aurosil Plus 5000 cst) on in vitro growth of common microorganisms causing endophthalmitis	Staphylococcus aureus, Staphylococcus epidermidis, Pseudomonas aeruginosa, MDR strain of Klebsiella pneumoniae, Escherichia coli, Candida albicans, and Aspergillus flavus	CFU number	5	III	Very low
Okonkwo et al. [26]	2018	Case series	5000 cSt	To report the long-term outcome of the management of a series of culture proven post pars plana vitrectomy endophthalmitis in which the infective agent was in the silicone oil used as an endotamponade	Burkholderia cepacia	BCVA e and Anatomical outcomes	4	III	Low

Steinmetz et al. [27]	2018	Case series	5000 cSt	To describe 2 cases of endophthalmitis successfully treated with an office injection of intravitreal antibiotics.	Not Applicable	BCVA, IOP, Anatomical outcomes	5	III	Very Low
Tayyib et al. [28]	1997	Case series	Not reported	To assess the onset of sterile endophthalmitis in silicone oil filled eyes	Not Applicable	Postoperative complication rate, incidence of endophthalmitis after vitrectomy and silicone oil tamponade	5	III	Very Low
Goel et al. [21]	2015	Case report	1000 cSt	To report the first case of multidrug-resistant endophthalmitis following pars plana vitrectomy in a silicone oil-filled eye	Pseudomonas Aeruginosa	At 1 month there was an attached retina and resolved peri phlebitis	5	III	Very Low

BCVA: Best Corrected Visual Acuity; cSt: Centi Stokes; CFU: colony-forming unit; SO: Silicone Oil; IOP: Intraocular Pressure; MDR: multidrug resistant. The level and the strength of evidence was defined according to the Oxford Centre for Evidence- Based Medicine (OCEM) 2011 guidelines and the Scottish Intercollegiate Guideline Network (SIGN) assessment system for individual studies as implemented for Preferred Practice Patterns by the American Academy of Ophthalmology respectively [14,15]. The quality of evidence based on the Grading of Recommendations Assessment, Development and Evaluation (GRADE) system was also assessed [16].
