# Supplementary material for: The Role of Silicone Oil in the Surgical Management of Endophthalmitis: A Systematic Review
Source: J Clin Med. 2022 Sep 16;11(18):5445. doi: 10.3390/jcm11185445 (PMC9505397; doi:10.3390/jcm11185445)
Supplement: Supplementary file 1 [file jcm-11-05445-s001.zip › jcm-1849201-supplementary-Table S2.pdf]

Table S2. Does Silicone oil injection after vitrectomy improve visual and anatomical outcomes?

Author	Year	Study Design	Sample	Groups	Purpose	Anatomical outcomes	Visual outcomes	Level	Strength	Grade
Dave et al. [29]	2017	Noncomparative, retrospective case series	93	Group 1(concurrent RD), Group 2 (Delayed RD).	To analyze the treatment outcomes in patients with endophthalmitis and concurrent or delayed-onset retinal detachment managed with pars plana vitrectomy, intravitreal antibiotics, and silicone oil.	Complete retinal reattachment rate was 73.7% in Group 1 and 98.5% in Group 2.	Visual acuity of 20/400 or better rate was 30.0% in Group 1 and 39.7% in Group 2	4	III	Low
Hudieb et al. [30]	2012	Comparative Case series	34	Group 1, 2 and 3 treated initially without silicone oil, group 4 treated initially with silicone oil.	To evaluate the role and outcomes of PPV and oil injection in the treatment of infectious endophthalmitis.	In 22 patients (group 1, 2 and 3) (55%) needed further surgery, either for persistent infection or retinal detachment 12 patients (group 4) treated at first with silicone oil had a rapid control of the infectious process and better anatomical results with this procedure only.	Final visual acuity was better in the silicone oil groups (group 3 and 4) than in the non-silicone groups (group 1 and 2).	4	III	Very low
Olgun et al. [16]	2018	Case Report	2	none	To report cases of Endophthalmitis after Xen Gel stent implantation treated with PPV and SOI	Infection control and attached retina was obtained in both cases.	Final visual acuity was hand motion in Case 1 and 20/400 in Case 2	5	III	Very low
Baxter et al. [17]	2015	Case Report	1	none	To report a case of S. Mitis endophthalmitis treated first with PPV and IOAB, then SOI.	At postoperative Month 1 attached retina with improved retinal perfusion on fluorescein angiography was observed.	At postoperative Month 1 BCVA was 20/200	5	III	Low
Aras et al. [31]	2001	Case Series	6	none	To investigate the use of silicone oil in the patients who had undergone vitrectomy for the treatment of endophthalmitis associated with retinal detachment	Final retinal reattachment and treatment of endophthalmitis was achieved in 5 eyes at the end of follow-up	Final visual acuity was 20/40 in 1 case, counting fingers in 4 cases and no light perception in 1 case.	4	III	Low
Bali et al. [32]	2003	Comparative Case Series	34	(Group 1): PPV+ IOAB. (Group 2) PPV+IOAB; PPV (group 3) PPV+IOAB; PPV+IOAB+SOI. (Group 4), PPV+IOAB+SOI	To evaluate the role of PPV and silicone oil injection in the treatment strategy of severe endophthalmitis.	In 22 patients (group 1, 2 and 3) (55%) needed further surgery, either for persistent infection or retinal detachment 12 patients (group 4) treated at first with silicone oil had a rapid control of the infectious process and better anatomical results with this procedure only.	Final visual acuity was better in the silicone oil groups (group 3 and 4) than in the non-silicone groups (group 1 and 2).	4	III	Very low
Pinarci et al. [33]	2013	Case Series	8	none	To report the role of early vitrectomy and silicone oil tamponade in acute endophthalmitis following intravitreal injection.	There was no retinal detachment or phthisis bulbi during the follow-up period (1–4 years)	BCVA at final follow-up was 0.05 in two patients (25 %), 0.1 in three patients (37.5 %),0.3 in two patients (25 %), and 0.8 in one patient (12. %).	4	III	Low

Farouk et al. [34]	2017	Comparative Case Series	26	Group 1 in which vitrectomy was done without silicon oil and group 2 in which vitrectomy was done with silicon oil. All cases were followed for 6 months.	To evaluate the outcomes of vitrectomy with and without silicone oil injection for the treatment of infectious endophthalmitis after cataract surgery when the retina is severely affected	Four cases (19 %) suffered from persistence of infection after vitrectomy and 3 cases (14.2 %) had postoperative retinal detachment in group 1. These complications were not reported in any case of group 2.	In Group 1 the mean logMAR visual acuity was 2.09 ± 0.82, in Group 2 the mean logMAR visual acuity was 2.04 ± 0.75	4	III	Low
Yan et al. [35]	2008	Case series	18	none	To explore the effects of vitrectomy combined with silicone oil injection in the treatment of traumatic endophthalmitis without retinal detachment and analyze the relative factors.	There was no retinal detachment or ocular atrophy	The postoperative visual acuity ranged from light perception to 0.8. The visual acuity increased in 15 eyes (83%), and was stable in 3 eyes (17%)	4	III	Low
Lin et al. [52]	2011	Retrospective case series	62	Patients were divided in groups according to the VA at presentation. Group A (12 cases; VA = LP) and group B (50 cases; VA>LP).	Authors investigated initial ocular conditions, surgical management and outcomes of PTE patients and analyzed their relationship in order to find the necessary management for different patients' conditions.	Initial VA, preventive scleral buckling and silicone oil tamponade may be good predictors of anatomic outcome.	In conclusion, for PTE, 69.4% (43/62) of the eyes with a good final visual outcome (VA >/= HM) was successfully managed with PPV, six of them underwent silicone oil injection.	4	III	Low
Khaqan et al. [46]	2017	Prospective study	112	In group 1 patients undergoing PPV with endotamponade (silicon oil) Group 2 patients undergoing PPV without endotamponade.	To evaluate the anatomical and functional outcomes of pars plana vitrectomy (PPV) in acute postoperative endophthalmitis with or without endotamponade.	23 (76.66%) patients out of 30 who underwent PPV only (Group 2) showed retinal detachment within first four weeks of follow up, while among 30 patients of Group 1 who underwent PPV with endotamponade, no patient showed retinal detachment in first four weeks post operatively	76 (92.68%) participants showed improved vision (6/36-6/60) in Group 1 and in Group 2 07 (23.33%) participants showed improved vision (6/36-6/60).	3	II	Low
Mohd-Ilham et al. [18]	2019	Case report	1	none	To report a case of a large subretinal abscess secondary to Klebsiella pneumoniae endophthalmitis in a pyelonephritis patient	The patient showed complete regression of the intraocular inflammation and subretinal abscess.	The patient regained her vision to 6/36.	5	III	Very Low
Mackiewicz et al. [24]	2000	Case report	1	none	the patient was treated with three vitrectomies. During the third vitrectomy the retinal detachment was repaired with circumferential buckle and silicone oil tamponade	If inflammatory changes of the retina are found during surgery, it seems advisable to administer silicone oil as a protection against detachment of the retina.	During the 2-year observation period, the visual acuity in the present case was 0.1	5	III	Very Low
Kapoor et al. [47]	2012	interventional consecutive retrospective study	30	Group 1 (n = 14) PPV + 1000 centistoke silicone oil tamponade for 12 weeks; Group 2 (n = 16) PPV + 1000 centistoke	to evaluate the efficacy of early vitrectomy with adjunctive silicone oil to treat endophthalmitis.	Additional surgery was required in 3% (1/30) in the study group.	At 9 months', 73% of all patients (22/30) achieved best corrected visual acuity (BCVA) of 20/40 or better.	3	II	Low

				silicone oil tamponade for 24 weeks.						
Kaynak et al. [48]	2003	Retrospective study	56	Group 1 (n = 24) eyes core vitrectomy; Group 2 (n = 28) eyes total PPV, encircling band, silicone tamponade, and endolaser	To evaluate the results of 2 surgical techniques in eyes with postoperative endophthalmitis.	The number of additional procedures was significantly less, and the rate of surgical success was significantly higher in Group 2 than in Group 1 (P<.01).	There was no statistically significant difference between the 2 groups in final visual acuity (P>.05).	3	II	Low
Verma et al. [36]	2017	Case series	9	none	Describes nine different real–world scenarios of endophthalmitis responding to intravitreal antibiotics alone and cases requiring intraocular lens removal, radical vitrectomy with hyaloid peeling, base dissection, and silicone oil	Not reported for all patients	Not reported for all patients	5	III	Very Low
Krėpštė et al. [19]	2013	Case report	1	none	To present a case of meningitis with bilateral endogenous bacterial pan ophthalmitis in a previously healthy individual.	Right eye was enucleated, two weeks after the removal of silicone oil the left eye suffered hypotony and subsequent phthisis bulbi.	At 13 weeks (BCVA) of the left eye was 0.07, Two weeks after the removal of silicone oil, visual acuity decreased to light perception.	5	III	Low
Nagpal et al. [43]	2012	Prospective Randomized study	129	Group 1 (n = 65) eyes, which underwent vitrectomy alone, were compared with group 2 (n = 64) eyes, in whom complete PPV with SOI was done.	To compare outcomes of pars plana vitrectomy (PPV) with and without silicone oil injection (SOI) in surgical management of endophthalmitis.	Rate of retinal detachment was 6.2% in group 2 as compared with 25.5% in group 1. Groups 1 and 2 required additional subsequent procedures in 27 eyes (41.54%) and 5 eyes (7.8%), respectively (P < 0.0001).	Mean best corrected visual acuity improvement was 0.867 ± 1.13 and 1.140 ± 0.88 in groups 1 and 2, respectively (P < 0.005). In the posttraumatic subgroup, difference between groups 1 and 2 in mean change in best corrected visual acuity was statistically significant (0.580 ± 1.10 and 1.132 ± 0.8 respectively, P < 0.05).	2	II	Moderate
Cakir et al. [37]	2009	Case series	8	none	to review microbiologic and medical records of eight cases of endophthalmitis caused by Fusarium species after cataract surgery.	All patients underwent multiple vitrectomies with silicone oil injections. One patient with corneal involvement underwent evisceration despite a variety of treatments. One patient with unregulated diabetes was pre phthisical without recurrence of infection. The final visual acuity of patients was between light perception and 20/100.	The final visual acuity of patients was between light perception and 20/100.	4	III	Low
Azad et al. [44]	2003	prospective randomized controlled study	24	Group 1: core vitrectomy<br>Group 2: complete vitrectomy with silicone oil endotamponade	to compare core vitrectomy with complete vitrectomy and silicone oil in posttraumatic endophthalmitis	Four (33.33%) of the 12 patients in group 1 developed retinal detachment following vitrectomy.<br>In group 2 Complete vitrectomy ensures the complete removal of vitreous membranes and prevents fibrous proliferation and tractional detachment	In group 1 41.66% of patients (5/12) achieving a useful visual outcome (≥20/400) and only one patient achieving a final visual acuity ≥20/200.<br>In group 2, 75% of patients (9/12) had a visual outcome better than 20/400 (P=0.07) and 58.3% (7/12) achieved a visual acuity greater than 20/200 (P=0.02)	3	II	Moderate

Siqueira et al. [49]	2009	Retrospective study	35	Group 1: intravitreal antibiotic injection, associated with topical and oral antibiotics<br>Group 2: vitrectomy with intravitreal antibiotic injection and silicone oil injection	To evaluate the outcomes of pars plana vitrectomy and silicone oil injection for the treatment of infectious endophthalmitis	Group 1: Six patients (25%) had retinal detachment during the first month of follow-up and also required PPV and SOI<br>Group 2: 2 patients (n=11), all of them had controlled infection on the first procedure. In one case (9.09%), a severe proliferative vitreoretinopathy (PVR) induced loss of vision (NLP)	Group 1: Nine patients (37.5%) had worsening of visual acuity, 10 patients (41.6%) improved and 5 patients (20.83%) did not change.<br>Group 2: One patient (9.09%) showed worsening of visual acuity, 5 patients (45.45%) improved and 5 patients (45.45%) did not change	3	II	Moderate
Wang et al. [50]	2011	Retrospective study	36	Group 1: 4 eyes without obvious retinal damage with BSS<br>Group 2: Sixteen eyes that had mild retinal damage filled with C3F8 gas<br>Group 3: 16 eyes with serious retinal damage were treated with silicone oil.	To study the criterion-reference of endotamponade in pars plana vitrectomy for metallic intraocular foreign body (MIOFD) associated with endophthalmitis	Group 1: There was no postoperative complication<br>Group 2: Only 2 cases occurred postoperative retinal detachment<br>Group 3: higher incidence of postoperative complications (18,8% retinal detachment, 25% ocular Hypertension, 31,3% needed secondary surgical treatment)	Group1: The visual acuity (VA) was improved<br>Group 2: postoperative VA improved in 10 eyes (62.5%), 4 eyes (25.0%) remained unchanged, and 2 eyes (12.5%) decreased.<br>Group 3: Postoperative VA of 9 eyes (56.3%) improved, 3 eyes (18.8%) remained unchanged, and 4 eyes (25.0%) decreased	3	II	Moderate
Do et al. [45]	2014	randomized controlled trial	108	Group 1 (53) standard PPV + IVT antibiotics.<br>Group 2 (55) standard PPV + IVT antibiotics + silicone oil	to compare treatment outcomes with and without silicone oil tamponade in patients undergoing (PPV for severe BEE	The anatomical result in Group 2 also had a trend of being better (Group 1, 64.2% versus Group 2, 80%; P=0.07).	The rate of VA improvement ≥ over baseline tended to be better in Group 2, 40% versus 22.6% of Group 1 (P=0.0521).	2	I-	Moderate
Jiang et al. [38]	2017	Retrospective study	121	none	To evaluate visual outcomes and identify prognostic factors after PPV surgery for traumatic endophthalmitis.	none	the use of silicone oil tamponade were not significant factors resulting in better BCVA	3	II	Moderate
Jin et al. [39]	2017	Retrospective study	107	none	to determine visual and anatomical outcomes of pediatrics patients with posttraumatic endophthalmitis following 23-gauge PPV combined with silicone oil endotamponade	Anatomical recovery 91.59%<br>Hypotony 0%<br>Silicone oil sustained eyes 1.87%<br>Silicone oil, low IOP 2.8 %<br>Atrophy 0.93%<br>Uncontrolled inflammation 1.87%<br>Evisceration in subsequent procedure 2.8%<br>Evisceration in primary procedure 0%	BCVAs were not only favorable, but also often better than those predicted by OTS.	4	III	Moderate
Lu et al. [87]	2019	Retrospective study	98	Group 1: only IV antibiotics (38)<br>Group 2: IV antibiotics + PPV (30)<br>Group 3: IV antibiotics + PPV + SO (27)	to evaluate the prognostic factors associated with visual outcomes in the salvageable eyes with post traumatic endophthalmitis between 2008 and 2015	The silicone oil group had fewer repeated intravitreal injections than the group without oil tamponade	The number of intravitreal injections were independently associated with poor visual outcome.	4	III	Moderate

				Group 4: intracameral antibiotics (3)					
Won et al. [25]	2013	case report	1	none	to report a case of acute postoperative endophthalmitis caused by vancomycin-resistant Staphylococcus hominis, treated with intraocular lens removal, and silicone oil tamponade	At 3 months, the retina was attached.	At 3 months, the visual acuity of the silicone oil-treated eye was 20/400.	5	III	Very Low
Siu et al. [22]	2015	case report	1	none	to report a case of endogenous endophthalmitis from K. pneumoniae.	At 3 months retinal re-detachment and reoperation.	At three months after the second operation BCVA 6/60.	5	III	Very Low
Zhang et al. [40]	2015	retrospective study	21	none	to evaluate the surgical efficacy and timing of 23-G vitrectomy for acute endophthalmitis following cataract surgery, and to determine when silicone oil tamponade and intraocular lens (IOL) removal are indicated during vitrectomy for endophthalmitis.	In all patients, the surgery resolved the endophthalmitis, one patient experienced recurrence of endophthalmitis 2 months after vitrectomy.<br><br>Two patients required primary silicone oil tamponade.<br><br>In 2 other patients, retinal detachment occurred with subsequent vitrectomy and silicone oil tamponade.	Two patients (9.5%) had BCVA >0.05 before treatment, 14 patients (66.7%) overall had BCVA >0.05 after treatment.<br><br>The difference was significant ($\chi 2$ = 15.003, p = 0.002).	4	III	Low
Chon et al. [23]	2017	case report	1	none	To report successful management of late-onset Streptococcus mitis endophthalmitis treated by vitrectomy, panretinal photocoagulation (PRP) and silicone oil tamponade	One month after the surgery, intraocular inflammation was stabilized.	visual acuity was improved from light perception to 20/200	5	III	Very Low
Yospaiboon et al. [41]	2018	Retrospective study	417	none	To determine factors affecting visual outcomes after treatment of infectious endophthalmitis. Methods of treatment were medical treatment (18.71%) and surgical treatment (81.29%), including pars plana vitrectomy with or without silicone oil tamponade (62.59%) and destructive surgery (18.71%).	none	After treatment, visual improvement was noted in 44.6%, stable vision in 18.47%, and worse vision in 36.93%.<br>Factors associated with improved visual outcomes were types of endophthalmitis, causative organisms, and initial visual acuity before treatment.	5	III	Low
Yospaiboon et al. [42]	2018	Retrospective study	45	none	To evaluate visual outcomes and possible predictive factors in the treatment of infectious endophthalmitis caused by *Streptococcus* species.	none	Nine patients (20%) had improved vision after treatment. The only predictive factor associated with improved visual outcomes was vitrectomy within 3 days. Medical treatment and PPV with antibiotics demonstrated more improved	4	III	Low

					Methods of treatment were medical treatment (18.71%) and surgical treatment (81.29%), including pars plana vitrectomy with or without silicone oil tamponade (62.59%) and destructive surgery (18.71%).		visual outcomes than PPV with antibiotics and silicone oil tamponade, but the difference was not statistically significant (*P*=0.072)			
Zhou et al. [51]	2020	retrospective study	22	Primary PPV+ SO:18 eyes Primary PPV +C3F8: 4 eyes	To explore the traumatic endophthalmitis in young children and the outcomes of pars plana vitrectomy	Five patients had retinal detachment (RD) within 3-4d of initial presentation. Four patients had traction RD after the second PPV, as a complication of surgery. Four patients exhibited band-shaped degeneration of the cornea during follow-up after the third operation. The final IOP was 8.9±1.8 mm Hg	The final BCVAs were 20/200 or better in five patients, two patients could count fingers, eight patients could detect hand movement, one patient had light perception and one patient had no light perception. Final BCVAs were not available for three patients. Whose (66.7%) had retinal injury exhibited worse BCVA (*P*=0.019, Fisher's exact test). Eyes underwent SO tamponade exhibited worse final BCVA than that with C3F8 in the primary PPV (*P*=0.026, Fisher's exact test).	4	III	Low

RD: Retinal Detachment; PPV: Pars Plana Vitrectomy; SO: Silicone Oil; SOI: Silicone Oil Injection; VA: Visual Acuity; BCVA: Best Corrected Visual Acuity; IOAB: Intraocular Antibiotic; IOP: Intraocular Pressure; MIOFD (Metallic Intraocular Foreign Bodies; HM: Hand Motion; IVT: Intravitreal Injection Therapy; BEE (endogenous bacterial endophthalmitis); OTS: Ocular Trauma Scores. The level and the strength of evidence was defined according to the Oxford Centre for Evidence- Based Medicine (OCEM) 2011 guidelines and the Scottish Intercollegiate Guideline Network (SIGN) assessment system for individual studies as implemented for Preferred Practice Patterns by the American Academy of Ophthalmology respectively [14,15]. The quality of evidence based on the Grading of Recommendations Assessment, Development and Evaluation (GRADE) system was also assessed [16].
