# Supplementary material for: The Role of Silicone Oil in the Surgical Management of Endophthalmitis: A Systematic Review
Source: J Clin Med. 2022 Sep 16;11(18):5445. doi: 10.3390/jcm11185445 (PMC9505397; doi:10.3390/jcm11185445)
Supplement: Supplementary file 1 [file jcm-11-05445-s001.zip › jcm-1849201-supplementary-Table S3.pdf]

Table S3. Risk of Bias (ROB) according to Revised Cochrane risk-of-bias tool for randomized clinical trials.

Author	Year	Study Design	Outcome to assess	Randomization process	Effect of assignment to intervention	Missing outcome data	Measurement of the outcome	Selection in reported results	Overall ROB judgement
Khaqan et al. [46]	2017	Individually randomized parallel-group trial	to assess the effect of assignment to PPV+ SOI	Some concerns	Some concerns	Low risk	Low risk	High risk	High risk of bias
Nagpal et al. [43]	2012	Individually randomized parallel-group trial	to assess the effect of assignment to PPV+ SOI	Low risk	Low risk	Low risk	Low risk	Low risk	Low risk of bias
Azad et al. [44]	2003	Individually randomized parallel-group trial	to assess the effect of assignment to PPV+ SOI	Some concerns	Low risk	Low risk	Low risk	Some concerns	Some concerns
Do et al. [45]	2014	Individually randomized parallel-group trial	to assess the effect of assignment to PPV+ SOI	Low risk	Low risk	Low risk	Low risk	Low risk	Low risk of bias

The development of the RoB 2 tool was supported by the MRC Network of Hubs for Trials Methodology Research (MR/L004933/2- N61), with the support of the host MRC ConDuCT-II Hub (Collaboration and innovation for Difficult and Complex randomized controlled Trials In Invasive procedures - MR/K025643/1), by MRC research grant MR/M025209/1, and by a grant from The Cochrane Collaboration.
