# Supplementary material for: The Role of Silicone Oil in the Surgical Management of Endophthalmitis: A Systematic Review
Source: J Clin Med. 2022 Sep 16;11(18):5445. doi: 10.3390/jcm11185445 (PMC9505397; doi:10.3390/jcm11185445)
Supplement: Supplementary file 1 [file jcm-11-05445-s001.zip › jcm-1849201-supplementary-Table S4.pdf]

Table S4. A detailed Newcastle-Ottawa Scale of each included retrospective study

				Selection				Comparability		Outcome			
**Author**	**Year**	**Study Design**	**Sample**	**Representativeness of exposed cohort**	**Selection of non-exposed cohort**	**Ascertainment of exposure**	**Demonstration that outcome of interest was not present at start of study**	**Adjust for the most important risk factors**	**Adjust for other risk factors**	**Assessment of outcome**	**Follow-up length**	**Loss to follow-up rate**	**Total quality score**
Lin et al. [52]	2011	Retrospective study	62	1	0	1	1	1	0	0	1	1	6
Kapoor et al [55].	2012	Retrospective study	30	1	0	1	1	0	0	0	1	1	5
Kaynak et al.[48]	2003	Retrospective study	56	1	0	1	1	1	0	0	1	0	5
Siqueira et al. [49]	2009	Retrospective study	35	1	1	1	1	1	0	1	1	0	7
Wang et al. [50]	2011	Retrospective study	36	1	1	1	1	1	0	1	0	1	7
Zhou et al. [51]	2020	Retrospective study	22	1	1	1	1	0	0	0	0	1	5

Thresholds for converting the Newcastle-Ottawa scales to AHRQ standards (good, fair, and poor):

Good quality: 3 or 4 stars in selection domain AND 1 or 2 stars in comparability domain AND 2 or 3 stars in outcome/exposure domain

Fair quality: 2 stars in selection domain AND 1 or 2 stars in comparability domain AND 2 or 3 stars in outcome/exposure domain

Poor quality: 0 or 1 star in selection domain OR 0 stars in comparability domain OR 0 or 1 stars in outcome/exposure domain
