# Supplementary material for: The Role of Silicone Oil in the Surgical Management of Endophthalmitis: A Systematic Review
Source: J Clin Med. 2022 Sep 16;11(18):5445. doi: 10.3390/jcm11185445 (PMC9505397; doi:10.3390/jcm11185445)
Supplement: Supplementary file 1 [file jcm-11-05445-s001.zip › jcm-1849201-supplementary-Table S5.pdf]

Table S5 JBI Critical Appraisal Checklist for Case series

JBI checklist questions	Okonkwo et al. 2018 [26]	Steinmetz et al. 2018 [27]	Tayyb et al. 1997 [28]	Dave et al 2017 [29]	Hudieb et al. 2012 [30]	Aras et al. 2001 [31]	Bali et al. 2003 [32]	Pinarci et al. 2013 [33]	Farouk et al. 2017 [34]	Yan et al. 2008 [35]
Were there clear criteria for inclusion in the case series?	Yes	Yes	Yes	Yes	Yes	Yes	Yes	Yes	Yes	Yes
Was the condition measured in a standard, reliable way for all participants included in the case series?	Yes	Yes	Yes	Yes	Yes	Yes	Yes	Yes	Yes	Yes
Were valid methods used for identification of the condition for all participants included in the case series?	Yes	Yes	Yes	Yes	Yes	Yes	Yes	Yes	Yes	Yes
Did the case series have consecutive inclusion of participants?	Yes	Yes	Unclear	Yes	Yes	Yes	Yes	Unclear	Yes	Yes
Did the case series have complete inclusion of participants?	Yes	Yes	Unclear	Yes	Yes	Yes	Yes	Unclear	Yes	Yes
Was there clear reporting of the demographics of the participants in the study?	Yes	Yes	No	Yes	No	No	No	Yes	Yes	Yes
Was there clear reporting of clinical information of the participants?	Yes	Yes	Yes	Yes	Yes	Yes	Yes	Yes	Yes	Yes
Were the outcomes or follow up results of cases clearly reported?	Yes	Yes	Yes	Yes	Yes	Yes	Yes	Yes	Yes	Yes
Was there clear reporting of the presenting site(s)/clinic(s) demographic information?	N/A	N/A	N/A	N/A	N/A	N/A	N/A	N/A	N/A	N/A
Was statistical analysis appropriate?	No	No	No	Yes	Yes	No	Yes	No	Yes	No

Table S5 (Continued)

JBI checklist questions	Verma et al. 2017 [36]	Cakir et al. 2009 [37]	Jiang et al. 2021 [38]	Jin et al. 2017 [39]	Zhang et al. 2015 [40]	Yospaiboon et al. 2018 [41]	Yospaiboon et al. 2018 [42]
Were there clear criteria for inclusion in the case series?	Yes	Yes	Yes	Yes	Yes	Yes	Yes
Was the condition measured in a standard, reliable way for all participants included in the case series?	Yes	Yes	Yes	Yes	Yes	Yes	Yes
Were valid methods used for identification of the condition for all participants included in the case series?	Yes	Yes	Yes	Yes	Yes	Yes	Yes
Did the case series have consecutive inclusion of participants?	No	Yes	No	Yes	No	Yes	Yes

Did the case series have complete inclusion of participants?	Unclear	Yes	Unclear	Yes	No	Yes	Yes
Was there clear reporting of the demographics of the participants in the study?	No	Yes	No	No	No	Yes	Yes
Was there clear reporting of clinical information of the participants?	Yes	Yes	Yes	Yes	Yes	Yes	Yes
Were the outcomes or follow up results of cases clearly reported?	Yes	Yes	Yes	Yes	Yes	Yes	Yes
Was there clear reporting of the presenting site(s)/clinic(s) demographic information?	N/A	N/A	N/A	N/A	N/A	N/A	N/A
Was statistical analysis appropriate?	No	No	Yes	Yes	Yes	Yes	Yes
