# Supplementary material for: The Role of Silicone Oil in the Surgical Management of Endophthalmitis: A Systematic Review
Source: J Clin Med. 2022 Sep 16;11(18):5445. doi: 10.3390/jcm11185445 (PMC9505397; doi:10.3390/jcm11185445)
Supplement: Supplementary file 1 [file jcm-11-05445-s001.zip › jcm-1849201-supplementary-Table S6.pdf]

Table S6. JBI Critical Appraisal Checklist for Case Reports

JBI checklist questions	Goel et al. 2015 [21]	Olgun et al. 2018 [16]	Baxter et al. 2015 [17]	Mohd-Ilham et al. 2019 [18]	Mackiewic et al. 2000 [24]	Krėpštė et al. 2013 [19]	Won et al. 2013 [25]	Siu et al. 2015 [22]	Chon et al. 2017 [23]	Yoon et al. 2003 [20]
Were patient's demographic characteristics clearly described?	No	No	No	No	Yes	No	No	No	No	No
Was the patient's history clearly described and presented as a timeline?	Yes	Yes	Yes	Yes	Yes	Yes	Yes	Yes	Yes	Yes
Was the current clinical condition of the patient on presentation clearly described?	Yes	Yes	Yes	Yes	Yes	Yes	Yes	Yes	Yes	Yes
Were diagnostic tests or assessment methods and the results clearly described?	Yes	Yes	Yes	Yes	Yes	Yes	Yes	Yes	Yes	Yes
Was the intervention(s) or treatment procedure(s) clearly described?	Yes	Yes	Yes	Yes	Yes	Yes	Yes	Yes	Yes	Yes
Was the post-intervention clinical condition clearly described?	Yes	Yes	Yes	Yes	Yes	Yes	Yes	Yes	Yes	Yes
Were adverse events (harms) or unanticipated events identified and described?	No	No	No	No	No	Yes	No	Yes	No	No
Does the case report provide takeaway lessons?	Yes	Yes	Yes	Yes	Yes	Yes	Yes	Yes	Yes	Yes
